# Diet and physical activity behaviors: how are they related to illness perceptions, coping, and health-related quality of life in young people with hereditary cancer syndromes?

**DOI:** 10.1007/s10865-024-00489-z

**Published:** 2024-04-20

**Authors:** Camella J. Rising, Chloe O. Huelsnitz, Rowan Forbes Shepherd, William M. P. Klein, Alix G. Sleight, Catherine Wilsnack, Patrick Boyd, Alexandra E. Feldman, Payal P. Khincha, Allison Werner-Lin

**Affiliations:** 1 Clinical Genetics Branch, Division of Cancer Epidemiology and Genetics, National Cancer Institute, 9609 Medical Center Drive, Rockville, MD 20850 USA; 2 Behavioral Research Program, Office of the Associate Director, Division of Cancer Control and Population Sciences, National Cancer Institute, Rockville, MD USA; 3 Department of Physical Medicine & Rehabilitation, Cedars-Sinai Medical Center, Los Angeles, CA USA; 4 Steve Hicks School of Social Work, University of Texas at Austin, Austin, TX USA; 5 USC Chan Division of Occupational Science and Occupational Therapy, Los Angeles, CA USA; 6 School of Social Policy and Practice, University of Pennsylvania, Philadelphia, PA USA

**Keywords:** Adolescents and young adults, Cancer, Diet, Physical activity, Health-related quality of life

## Abstract

Individuals with inherited cancer syndromes, such as Li-Fraumeni syndrome (LFS), may be motivated to adopt health-protective behaviors, such as eating more fruits and vegetables and increasing physical activity. Examining these health behaviors among young people with high lifetime genetic cancer risk may provide important insights to guide future behavioral interventions that aim to improve health-related quality of life (HRQOL). We used a self-regulatory framework to investigate relationships among diet and physical activity behaviors and psychosocial constructs (e.g., illness perceptions, coping, HRQOL) in adolescents and young adults (AYAs; aged 15–39 years) with LFS. This longitudinal mixed-methods study included 57 AYAs aged 16–39 years at enrollment), 32 (56%) of whom had a history of one or more cancers. Participants completed one or two telephone interviews and/or an online survey. We thematically analyzed interview data and conducted regression analyses to evaluate relationships among variables. AYAs described adopting healthy diet and physical activity behaviors to assert some control over health and to protect HRQOL. More frequent use of active coping strategies was associated with greater reported daily fruit and vegetable intake. Greater reported physical activity was associated with better quality of psychological health. Healthy diet and physical activity behaviors may function as LFS coping strategies that confer mental health benefits. Clinicians might emphasize these potential benefits and support AYAs in adopting health behaviors that protect multiple domains of health. Future research could use these findings to develop behavioral interventions tailored to AYAs with high genetic cancer risk.

**Supplementary Information:**

The online version contains supplementary material available at 10.1007/s10865-024-00489-z.

## Introduction

Hereditary cancer syndromes (e.g., hereditary breast and ovarian cancer, Li-Fraumeni) predispose individuals to high cancer risk and potentially significant chronic physical and psychosocial health challenges (Barnett et al., 2022; Gopie et al., 2012). In situations of high genetic risk, individuals may be more fatalistic about cancer and less motivated to adopt healthier behaviors (Marteau & Weinman, 2006). However, past research suggests some individuals with hereditary cancer syndromes may be motivated to adopt health-protective behaviors, such as eating more fruits and vegetables and being more physically active (Rees et al., 2006; Spector, 2007). In studies of cancer survivors, healthy diet and physical activity behaviors have been associated with improved health-related quality of life (HRQOL) (Blanchard et al., 2008; Rock et al., 2022). Psychosocial factors, such as illness perceptions (e.g., perceived control, cancer worry), may influence whether individuals adopt and maintain these healthy behaviors (Durazo & Cameron, 2019; Séguin Leclair et al., 2021).

Theoretical linkages among illness perceptions, diet, physical activity, and HRQOL have not been examined in young people with early-onset hereditary cancer syndromes, including those without a prior cancer history. Moreover, past studies have not specifically investigated these health behaviors and psychosocial associations in adolescents and young adults (AYAs; aged 15 to 39 years) with Li-Fraumeni syndrome (LFS), the focus of this study. Adolescence and young adulthood is a period marked by significant cognitive, psychosocial, and emotional development as well as the establishment of lifelong health and health behavior trajectories, all of which may be disrupted by the threat and/or effects of cancer (Pugh & Fisher, 2018).

### The case of Li-Fraumeni syndrome

LFS is an inherited cancer syndrome associated with nearly 100% lifetime cancer risk from birth. The incidence of any cancer is 24 times that of the general population, with the highest comparative incidence in childhood through early adulthood (0–30 years). Predominantly caused by pathogenic germline variants in *TP53*, LFS-related cancers may develop across multiple organ systems (e.g., breast, brain, blood, bone, soft tissue) and individuals with LFS have a high risk of developing multiple primary cancers (de Andrade et al., 2021). Limited options for early detection and prevention include intensive multi-organ cancer screening (e.g., annual whole-body MRI, brain MRI) and risk-reducing mastectomy for females (Khincha et al., 2019; Kratz et al., 2017). AYAs with LFS often need psychosocial support to cope with complex and dynamic health challenges amid normative, but also potentially stressful, life transitions (e.g., developing autonomy, forming a family) (Forbes Shepherd et al., 2018; Werner-Lin, 2022). Given the chronic high threat of cancer and limited to no (for males) options for cancer prevention, eating healthfully and being physically active may serve as opportunities for all young people diagnosed with LFS, including those with and without a cancer diagnosis, to cope with LFS by taking actions that protect both physical and psychosocial health in the short and long term.

### Young people, cancer (risk), and health-protective behaviors

Past studies have shown that AYA cancer survivors are interested in receiving information about health-protective behaviors, especially regarding diet and physical activity, and believe that it is important to offer such information before, during, immediately after, and in post-treatment periods (Pugh et al., 2017). Healthy behaviors recommended for cancer risk reduction and better survivorship outcomes (e.g., cancer treatment outcomes, HRQOL) typically include eating patterns that emphasize fruits and vegetables (at least 1.5-2 cups fruit and 2.5-3 cups vegetables daily) and physical activity patterns that involve at least 150 min of weekly moderate-to-vigorous intensity physical activity (Rock et al., 2020, 2022). Yet, diet quality and physical activity of AYA cancer survivors are often suboptimal (Rabin et al., 2011; Skiba et al., 2021), which may be due to multiple factors such as cancer treatment effects (e.g., fatigue), lack of information, or low self-efficacy (Pugh et al., 2017; Pugh & Fisher, 2018; Skiba et al., 2021).

Whether AYAs with early-onset hereditary cancer syndromes, such as LFS, have these perspectives and diet and physical activity behaviors after or prior to a cancer diagnosis has not been reported in the literature. However, research focused on adult-onset syndromes (e.g., hereditary breast and ovarian cancer) (Rees et al., 2006) and adult LFS populations (Nees et al., 2022), suggest that AYAs with LFS might be motivated to adopt healthier behaviors after an LFS or LFS-related cancer diagnosis. Given that a nutritious diet and physical activity may improve physical and psychosocial health outcomes as well as strengthen and stabilize at-risk organ systems *prior to* a cancer diagnosis, AYAs with poor dietary intake or physical inactivity would miss these potential health benefits. Moreover, due to high risk of cancers at a young age and the distress of personal and family cancer diagnoses, AYAs with LFS may be more vulnerable to poor physical and psychosocial health and have different challenges engaging in healthy behaviors compared with younger-, older-, or same-age non-LFS peers (Sleight et al., 2022; Warner et al., 2016). Determining whether diet and physical activity behaviors function as coping strategies or influence HRQOL in the context of high genetic cancer risk could provide directions for future intervention research that aims to improve health outcomes among all AYAs with LFS, including those who are not cancer survivors but who experience complex LFS-related psychosocial health challenges.

### Examining diet and physical activity behaviors using self-regulatory frameworks

To guide our investigation into psychosocial associations of diet and physical activity behaviors, we used the common-sense model of self-regulation (CSM), a theoretical framework that has been widely applied in the context of cancer (Leventhal et al., 2016; Richardson et al., 2017), including genetic risk (Kelly et al., 2005; Marteau & Weinman, 2006). Based on the theoretical concepts and pathways of the CSM, AYAs who perceive LFS as an illness threat will form perceptions of LFS—*illness representations*—that influence their coping responses, which ultimately affect health outcomes. Cognitive illness representations comprise five distinct attributes: *illness identity* (labeling the illness and experiencing related symptoms) and perceptions of the *consequences*, *control*, *timeline*, and *causes* of the illness. These attributes, along with emotional illness representations (e.g., cancer worry), theoretically drive the selection of coping strategies (e.g., adopting health-protective behaviors) to manage health outcomes (e.g., quality of physical or psychological health). However, past studies conflict with regard to associations among these variables in cancer survivor populations (Durazo & Cameron, 2019; Séguin Leclair et al., 2021) and in the general population (Ferrer et al., 2013).

To our knowledge, this is the first study to use a self-regulatory framework to examine illness perceptions, diet, and physical activity in young people with a rare hereditary cancer syndrome. We posit that high, lifelong, multi-organ cancer risk and limited prevention options may heighten the perceived consequences of LFS and cancer worry as well as lessen perceived personal control over LFS. In this analysis, we were especially interested in exploring relationships among these illness perceptions, diet, physical activity, LFS coping strategies, and HRQOL. Exploring these linkages in AYAs with LFS may provide broader insights into the heuristics used by young people with hereditary cancer syndromes to process risk information, formulate representations of illness threats, and find coping responses that “fit” (Marteau & Weinman, 2006).

### Study aims

The goal of this study was to better understand diet and physical activity behaviors and the meaning of those behaviors for AYAs with LFS, including those with and without a prior cancer history. Our primary aim was to use qualitative interview data to investigate AYAs’ reported diet and physical activity behaviors and their perspectives on engaging in these behaviors in the context of high, lifelong cancer risk. Secondary aims of this study were (1) to use quantitative survey data to explore associations among AYAs’ reported diet and physical activity behaviors—specifically, amount of daily fruit and vegetable intake and level of physical activity—and illness representations, LFS coping strategies, and quality of physical and psychological health and (2) to evaluate congruence between the qualitative and quantitative findings. Findings conflict regarding relationships among CSM constructs (e.g., Durazo & Cameron, 2019) and gaps exist in understanding health behaviors and how they relate to illness perceptions, coping, and HRQOL among AYAs. Thus, we posed the following research questions:

#### RQ1

To what extent is daily fruit and vegetable intake associated with prior cancer history, cancer worry, perceived control of LFS, perceived LFS consequences, LFS coping strategies, and perceived quality of physical and psychological health among AYAs with LFS?

#### RQ2

To what extent is level of physical activity associated with prior cancer history, cancer worry, perceived control of LFS, perceived LFS consequences, LFS coping strategies, and perceived quality of physical and psychological health?

## Methods

This analysis was nested in a larger longitudinal mixed methods behavioral study under the National Cancer Institute’s LFS study. The behavioral study used an embedded design (Creswell & Clark, 2017) in which quantitative data were embedded within a qualitative framework to address separate but complementary research questions regarding the lived experience of AYAs with LFS. Embedded mixed-methods approaches have been used to enrich understanding of nutrition-related behavior in cancer survivorship (Loeliger et al., 2021).

Given the formative nature of this study, our analysis was predominantly focused on qualitative data from two waves of interviews. To enrich our understanding of AYAs’ LFS-related experiences, perspectives, and responses to LFS, we complemented our qualitative analysis with an analysis of quantitative data from an online survey conducted concurrently with interviews at the second wave. Data collection and analysis was an iterative process, whereby findings from wave 1 interviews informed development of the wave 2 interview guide and the online survey. For example, the wave 1 interview guide elicited AYAs’ perspectives and reported health-protective behaviors in response to LFS broadly; thus, when developing the wave 2 interview guide, we added questions to elicit the *meaning* of the LFS-related health-protective behaviors AYAs reported in wave 1 interviews (e.g., How do you benefit from them? What purpose do they serve?). In addition, we included survey measures to assess reported health-protective behaviors (e.g., fruit and vegetable intake, physical activity) identified in wave 1 interview data, enabling methodological triangulation. In the report that follows, qualitative and quantitative results are presented separately, and data were mixed at the level of interpretation to clarify convergence and divergence between data sets. Study procedures were approved by the National Institute of Health IRB (NIH Protocol 11-C-0255, ClinicalTrials.gov; Identifier NCT01443468).

### Sample and recruitment

We recruited 57 participants enrolled in the IRB-approved LFS study. For this analysis, individuals were eligible to participate if they were (1) diagnosed with a pathogenic germline *TP53* variant and were (2) aged 15 to 39 years at the time of initial recruitment. Parental permission was obtained prior to enrollment of AYAs under age 18.

### Data collection

#### Qualitative interviews

Our interprofessional team of clinician-researchers developed semi-structured interview guides (see Electronic Supplementary Material 1) to examine participant perspectives on LFS-related personal health and well-being, health beliefs (e.g., illness control beliefs), and engagement in health behaviors. AYAs’ perspectives on these topics were assessed in wave 1 interviews (October 2019-March 2020) and/or wave 2 interviews (March-July 2021). In wave 2 interviews, those who also participated in wave 1 were asked to describe any changes in LFS-related personal health and well-being and engagement in health behaviors since wave 1 interviews. All wave 2 participants were asked about the effect of the COVID-19 pandemic on LFS-related health, well-being, and health behaviors. Interviews were conducted via telephone and audio recorded after obtaining participant consent. We transcribed interviews verbatim and removed all personally identifiable information.

#### Quantitative surveys

Eligible participants completed an online survey in Qualtrics between February and September 2021. After completing the online consent form, participants responded to items pertaining to illness representations, LFS coping strategies, health behaviors, and demographics. All qualitative and quantitative data were stored on password-protected computers.

### Survey measures

#### Daily fruit and vegetable intake

We measured reported daily fruit intake and daily vegetable intake with two items from the National Cancer Institute’s Food Attitudes and Behaviors Survey (FAB) (Yaroch et al., 2012) (e.g., “About how many cups of fruit [including 100% pure fruit juice] do you eat or drink each day?” 1=“none” to 7=“4 or more cups”).

#### Level of physical activity

We used items adapted from the FAB to measure reported level of physical activity (Yaroch et al., 2012). These items included, “In a typical week, how many days do you do any physical activity or exercise of at least moderate intensity, such as brisk walking, bicycling at a regular pace, and swimming at a regular pace?” (1=“0 days” to 8=“7 days”) and “On the days that you do any physical activity or exercise of at least moderate intensity, how long do you typically do these activities?” (open-ended text response). For respondents who reported ≥ 1 day(s) of at least moderate-intensity physical activity, we calculated level of physical activity by multiplying the reported number of days per week of at least moderate-intensity physical activity by the minutes of moderate-intensity physical activity typically done on those days.

#### Illness representations

We measured cognitive illness representations—LFS consequences and personal control of LFS—using two items from an adapted Brief Illness Perception Questionnaire (Brief IPQ) (Broadbent et al., 2006) (“I feel like I have control over my LFS” and “LFS affects my life”). These items were assessed separately as they are viewed as conceptually distinct (Séguin Leclair et al., 2021). Emotional illness representations (LFS concern, cancer risk concern, and frequency of cancer risk worry) were measured using one item from the adapted Brief IPQ (“I am concerned about my LFS”) and two items from an adapted Genetic Psychosocial Risk Instrument (GRPI) (Esplen et al., 2013) (e.g., “I worry often about my risk of getting cancer”). These three items were averaged and assessed as a single variable (α = .85). Illness representation items were measured on a 5-point Likert scale (1=“disagree strongly,” 3=“neither disagree nor agree,” and 5=“agree strongly”).

#### LFS coping strategies

In the interest of minimizing participant burden and optimizing survey length, we used an adapted, truncated Brief COPE inventory (Carver, 1997) to measure nine LFS coping strategies, active coping, acceptance, emotional support, humor, instrumental support, planning, religion, self-distraction, and venting. Items measured the reported extent to which participants used these strategies to cope with LFS in the past month, (e.g., “Taking action to try to make the situation better,” 1=“I haven’t been doing this at all” to 4=“I’ve been doing this a lot”). The nine subscales were each comprised of two items averaged together.

#### Health-related quality of life

We measured reported HRQOL in the past month using an adapted World Health Organization WHOQOL-BREF (World Health Organization, 1998), an instrument previously used to examine cancer survivor populations (Den Oudsten & Skevington, 2022). Items assessed perceived quality of physical health (e.g., “When I need to, I am able to get around”) and psychological health (*e*.*g*., “My life is meaningful”) on a 5-point Likert scale (1=“strongly disagree,” 3=“neither disagree nor agree,” and 5 = “strongly agree”). Quality of physical health was measured with seven items that were averaged together to calculate a physical health domain score (α = 0.86). Six items measured quality of psychological health and were averaged to calculate a psychological health domain score (α = 0.86). Mean domain scores were multiplied by four to make domain scores comparable with WHOQOL-100 scores (range = 0-100), the long-form assessment of HRQOL.

#### Demographics

We collected self-reported data on the following demographic characteristics: age, age at genetic testing, gender (male, female, genderqueer), race (American Indian/Alaska Native, Asian, Black/African American, Native Hawaiian/Other Pacific Islander, White), ethnic identity (Hispanic/Latino, non-Hispanic/Latino), educational attainment (< high school graduate, high school graduate, technical or vocational school, some college, ≥college graduate), prior cancer history (yes/no), and age at first primary cancer diagnosis.

### Data analysis

#### Thematic analysis

We used thematic analysis to analyze interview data (Clarke & Braun, 2014). To develop a preliminary codebook, our coding team used a hybrid inductive-deductive open coding strategy. *A priori* codes and operational definitions were based on several concepts taken from the CSM (Leventhal et al., 2016), including illness representations (e.g., perceived control, cancer worry), coping strategies, health behaviors, health beliefs, and perceptions of health and mental health status. The team discussed and resolved coding discrepancies to compile a final codebook with codes, definitions, and parameters for use. The first author thematically analyzed data by assigning conceptual codes to text in Dedoose version 9.0.85, grouping codes into categories on the basis of thematic salience, and identifying properties to define categories (Strauss & Corbin, 1998). The team verified the final themes and their properties to ensure analytic rigor.

#### Statistical analysis

RStudio version 1.4.1717 was used to calculate descriptive statistics and perform correlations and multiple linear regressions on psychosocial correlates of daily fruit/vegetable intake (fruit and vegetable intake scores were combined and assessed as a single variable), level of physical activity, illness representations, coping strategies, and HRQOL. Due to sample size constraints and the potential for differences by prior cancer history (LeMasters et al., 2013), we included all participants in regression models and controlled for, rather than stratified by, prior cancer history. We used standardized variables in regression models to calculate standardized coefficients (*β*) with 95% confidence intervals (CI). Statistical significance was set at *p* < .05.

## Results

### Sample

In total, 57 AYAs participated in this study, of whom 22 (39%) opted to complete an interview and the survey, 20 (35%) opted to only complete an interview, and 15 (26%) opted to only complete the survey. Of 57 participants, about two thirds (*n* = 38, 67%) completed wave 1 interviews, approximately half completed wave 2 interviews (*n* = 30, 53%), and nearly two thirds responded to the online survey (*n* = 37, 65%), which took less than 30 min to complete (median = 23 min). Wave 1 interviews averaged 69 min (range = 17–122 min) and wave 2 interviews averaged 78 min (range = 44–129 min). Participants had a mean age of 30 years (*SD* = 6.5 years, range = 17–40 years). Mean age at genetic testing was 23 years (*SD* = 7.5 years, range = 1–35 years). Participants were predominantly female (*n* = 41/57, 72% vs. male: *n* = 15/57, 26% vs. genderqueer: *n* = 1/57, 2%), White (*n* = 49/56, 86% vs. Asian: *n* = 2/56, 4% vs. multiracial: *n* = 5/56, 9% ), non-Hispanic/Latino (*n* = 53/56, 83% vs. Hispanic/Latino: *n* = 3/56, 5%), and college graduates (*n* = 32/49, 56% vs. < college graduate: *n* = 17/49, 23%). Most interview participants in wave 1 (*n* = 23/38, 61%) and wave 2 (*n* = 20/30, 67%) had one or more primary cancers, and about half of survey respondents reported a prior cancer history (*n* = 19/37, 51%). In the total sample, mean age at first primary cancer diagnosis was 22 years (*SD* = 9.8 years, range = 0.5–35 years). Electronic Supplementary Material 2 Table S1 provides demographic characteristics of the sample by participation mode.

### Perspectives on diet and physical activity behaviors

Our first aim was to use qualitative data to investigate AYAs’ perspectives on diet and physical activity behaviors in the LFS context. Most AYAs reported attentiveness to eating and being physically active for health or cancer risk reduction. They often described healthy diets as eating patterns that emphasize fruits and vegetables and that limit “processed foods,” fast foods, added sugars, and alcoholic beverages. AYAs also described a range of routine physical activities they participated in, such as walking, running, hiking, resistance training, stretching, yoga, Pilates, and martial arts.

We grouped themes pertaining to AYAs’ perspectives on diet and physical activity behaviors into two categories using the CSM as a guiding framework: (1) *striving for a sense of control over health* and (2) *engaging in diet and physical activity behaviors to protect HRQOL*. Illustrative quotes for each category are incorporated into thematic findings. Pseudonyms were used for all quotes presented. Age and prior cancer history reported next to quotes refer to the AYA’s age and cancer status at the time they were interviewed.

### Striving for a sense of control over health

Most AYAs described their diet and physical activity behaviors as actions they could take in their daily lives to assert some control over their health while living with largely unpreventable, high genetic cancer risk. Several mentioned a particularly intense drive to take charge of health behaviors in the period just following their LFS or LFS-related cancer diagnosis. However, some reported difficulty processing information about health-protective behaviors in this period due to cancer worry and lack of information. Jayne (age 30, no cancer history) shared: “When I first found out about my mutation, I really started digging into what I had control over… I immediately started looking at environmental factors… I went a little crazy when I first found out. And, so, I got a water purifier… I got a juicer.” Others, like Charlotte (age 38, no cancer history), mentioned that behavioral changes surrounding the time of her LFS diagnosis were fear-driven: “After the initial diagnosis, I think I was really scared for a period of time. I don’t even think I would eat cheese, you know? It was almost like every day, just kind of waiting for something horrible to happen.”

Many AYAs reported that, over time, they became less anxious about their health behaviors, recognizing that, while they provided a sense of control, they would not necessarily prevent cancer given their high genetic cancer risk. Chelsea (age 18, cancer survivor) shared that accepting her limited options for cancer control took time:

The only aspects of my health that I can control are what I eat [and] how I manage my activity. There’s no way that I would ever be able to prevent myself from… having cancer… That’s been something that I’ve had to work on over the years.

Like Chelsea, Mr. T (age 35, cancer survivor) delineated the difference between taking control of his health versus preventing cancer. Rather than framing his sense of control over LFS around the prevention of cancer, he relied on how “good” he looked and that guided his perceptions of health:

I’m very, very much in control of my health but very out of control of my cancer issue… When I eat certain foods… and when I exercise, I feel good. I look good. I’m generally healthy. But my thought is that those things cannot or are a lot less likely to affect whatever cancer is going to do to me.

Some AYAs shared that although health-protective behaviors provided opportunities to take action in the face of high lifetime cancer risk, the sense of control these behaviors provided was sometimes dampened by the apparent randomness of cancer development. Charlotte (age 38, no cancer history), for example, described how witnessing LFS-related cancers and mortality in her family tempered the intensity of her efforts to control her health through diet and physical activity.

When I first was confirmed that I had it [LFS], I kind of went extreme… I know that there are things you can do, but then I also know that it’s [cancer] totally random. I feel like my healthy cousins [that had LFS] are the ones that have passed away….

Being (re)diagnosed with cancer despite healthy lifestyle behaviors or otherwise good health status also influenced the extent to which AYAs felt in control. Artemis (age 38, multi-cancer survivor) explained that her second cancer diagnosis led to a loss of self-identity because she had put a great deal of effort into changing her health behaviors after her first cancer diagnosis:

I went through a little bit of an ego death with my second diagnosis, because I think after my first one, I was like, ‘Okay, if I do the medication and I eat the green juice and I do the thing and get the acupuncture and meditate on the hill and take the mushrooms, I’ll be okay.’ And then I got re-diagnosed doing quote, unquote, everything right.

Many AYAs mentioned the importance of feeling in control of rather than controlled by health behaviors. For example, Veronica (age 25, no cancer history) shared that feeling controlled by her diet behaviors would threaten life enjoyment: “I try to eat as much greens… and foods that help prevent cancer cells from growing… But I don’t let it control my life because, if I did, I think I would be miserable.” Other AYAs illustrated the psychological costs of feeling that health behaviors must be controlled due to the high cancer risks associated with LFS. George (age 36, cancer survivor) said: “I basically have this mentality that if I stop [exercising], I die.” He also shared that although he once enjoyed sweets as a child, he “stopped associating those types of things with pleasure” as he now believed sweets harm his body. These perspectives stand in stark contrast to the control beliefs of Mona (age 24, multi-cancer survivor) who said: “Doesn’t matter what I eat, doesn’t matter how much weight I lose, doesn’t matter what I do… [LFS] doesn’t go away.”

### Engaging in diet and physical activity behaviors to protect HRQOL

Several AYAs reported that LFS or their LFS-related cancer diagnosis motivated health behavior change or increased their awareness of the health benefits associated with engaging in healthy diet and physical activity behaviors. Cindy (age 30, cancer survivor) described behavior changes she made in response to her early-onset cancer as a silver lining:

Yes, it’s really sad that I got cancer and I got diagnosed with this mutation at a young age, but… now I know how to better take care of my body and take care of my health. Before I was diagnosed, I probably would stay up really late and ate whatever I want. Now, I try to go to bed before 11:00… Also, I try to eat healthy with vegetables, fruit, and meat. I try to have a balanced diet.

Many AYAs talked about the benefits of healthy diet and physical activity on their physical and psychological health. Some mentioned that these health behaviors were an important part of protecting their bodies from, as well as preparing their bodies for, a future cancer given their high genetic cancer risk. Jacob (age 29, no cancer history) said, for example: “My daily attitude is to be as healthy as I can physically… also [with] what I’m eating… Then, if anything were to arrive, I’m in the best state to deal with [cancer] and then hopefully, God willing, I can prevent it altogether.”

Other AYAs described the value of physical activity in cancer survivorship, including keeping their bodies strong during cancer treatments, helping them rehabilitate after treatments, and providing stress relief. Some, like Lauren (age 37, cancer survivor), described physical activity as therapeutic. She likened exercise to medicine, with the caveat that it benefited her mental health as long as she approached it with balance and self-compassion: “It’s really true that exercising is my mental health pill, but I’m not super religious about it either. If I skip a day, I don’t freak out. I used to get anxiety about not exercising.” Mattie (age 33, multi-cancer survivor) also remarked that activities, like yoga, served as ways to psychologically cope with the negative effects of multiple primary cancer diagnoses:

If I ever start to feel anxious or depressed, I get myself out of it pretty quickly with doing yoga or just sitting and being still and calm. And then I try to just focus on my breathing, and it really works.

Allan (age 28, no cancer history) offered a nuanced perspective on the therapeutic nature of diet and physical activity behaviors. With the support of mental health counseling, he noticed a positive feedback loop involving his mental health and healthy behaviors:

Dealing with the strength of my mental health has, in turn, given back to the physical aspects of my body. The better I feel mentally, the more inspired I am to be active and to eat healthy, and make sure I’m not staying in bed all day.

Healthy behaviors also helped AYAs cope with difficult thoughts and emotions due to the psychosocial burdens of LFS, such as worry, “scanxiety,” grief, and anger. Artemis (age 38, multi-cancer survivor), for example, shared that dietary changes were a part of her health “reset” around the time of her multiple, routine cancer screenings:

I set up a lot of things around nutrition, supplements, acupuncture to reset my system… I have little rituals I do around each one of my scans to recalibrate because it’s like you get scrambled like a paint can and you don’t feel great.

Although George (age 34, cancer survivor) recognized physical activity was not a replacement for “real therapy,” he described it as a strategy to cope with grief and anger after the loss of family members to LFS-related cancers: “I started [exercising] after that [family deaths] because I was having a lot of emotional, like angry, problems that arose from it. I basically got sat down [by family] and told, ‘you need to find like a healthy outlet for this’.”

Not all AYAs changed their health behaviors in response to LFS, and several described barriers to engaging in healthy behaviors, such as a lack of information about nutrition recommendations after LFS diagnosis, low motivation, and low self-efficacy. Mattie (age 33, cancer survivor) shared, for example, that experiencing multiple LFS-related cancers had not motivated her to change her eating patterns: “You would think that I would have it scared into me that I should eat better with having cancer a couple of times, but I just, and I don’t eat terrible, but I eat a lot of pizza and bread.” Rick (age 40, no cancer history) mentioned that lack of confidence in his ability to change behaviors may be what holds him back: “I just need to get a little more motivated and control things better, but I don’t have the willpower to do so.” Other AYAs mentioned external factors that hindered them from a healthier lifestyle, including social norms (e.g., drinking alcohol with peers), the time constraints of parenting and caregiving, and shifts in daily life due to the COVID-19 pandemic.

Short- and long-term effects of cancer and treatments also challenged some AYAs’ ability, comfort, enjoyment, or recovery from engaging in physical activity. Sophie (age 33, cancer survivor), for example, reported that pain due to past cancer treatments made it difficult to exercise as much as she preferred: “I don’t exercise a ton, but that’s just because I don’t feel like I can… That’s more of just like the chronic pain thing.” Silver (age 31, multi-cancer survivor) also described the role of treatment effects on her yoga practice, remarking that functional shifts and losses sometimes upset her mental health:

Some yoga poses I just cannot do [because] my chest muscles are on top of my breast. My body doesn’t go like that anymore and that can sometimes feel really difficult and triggering. I feel like most of the time yoga’s really good and relaxing for me. And then sometimes, I end up leaving really mad and upset about all the ways my body feels like it’s failed me.

### Relationships between health behaviors, illness representations, coping, and HRQOL

The second aim of this study was to use quantitative data to explore associations among AYAs’ reported daily fruit/vegetable intake, level of physical activity, and psychosocial predictors, namely illness representations, LFS coping strategies, and HRQOL. Seventeen of 37 AYAs (46%) reported consuming greater than or equal to 1–2 cups fruits and 12 of 37 (32%) reported consuming greater than or equal to 2–3 cups vegetables daily; therefore, under half and about one third met American Cancer Society recommendations for daily fruit and vegetable intake, respectively. Combined scores for reported daily fruit and vegetable intake indicated that AYAs consumed, on average, greater than or equal to 4 cups of fruits and vegetables daily (*M* = 7.51, *SD* = 2.57, median = 7, range = 4–13). Four AYAs reported 0 days of at least moderate-intensity activity per week and five had missing data; therefore, physical activity findings were based on reported level of physical activity for 28 respondents. The average reported amount of weekly moderate-intensity physical activity was about 130 min (*M* = 129.64 min, *SD* = 94.08 min, median = 105 min, range = 20–315 min). Under one third of AYAs (*n* = 8/28, 29%) reported greater than or equal to 150 min of moderate-intensity physical activity weekly, meeting American Cancer Society recommendations. Acceptance and self-distraction were coping strategies AYAs reported using with the greatest relative frequency (*M* > 2.5), followed by active coping and planning (*M* = 2.5). See Electronic Supplementary Material 3 Table S2 for descriptive statistics.

First, we visually examined histograms of each of the key variables in conjunction with the descriptive statistics. All variables were approximately normally distributed, with the exception of quality of physical and psychological health, for which there was a negative skew and high variability (e.g., quality of physical health responses ranged from 6 to 100, with *SD* = 21.74; quality of psychological health responses ranged from 6 to 94, with *SD* = 18.25). In line with our exploratory analytic aims, we then examined correlations between the key variables in our study (see Electronic Supplementary Material 4, Table S3). Daily fruit and vegetable intake was significantly and positively correlated with two forms of coping: active coping (*r* = .34, *p* = .046) and humor (*r* = .37, *p* = .025). Physical activity level was significantly and positively correlated with quality of physical health (*r* = .40, *p* = .036) and psychological health (*r* = .40, *p* = .034).

We then performed separate multiple linear regressions between each of the 13 predictor variables and each of the two outcomes, controlling for cancer history (see Electronic Supplementary Materials 5 and 6). Controlling for cancer history, the use of active coping (*β* = 0.34, *p* = .049, 95% CI 0.00-0.68, adjusted *R*^2^ = 0.06) and the use of humor (*β* = 0.37, *p* = .025, 95% CI 0.05–0.70, adjusted *R*^2^ = 0.09) were both associated with daily fruit/vegetable intake, such that greater use of active coping or greater use of humor in response to LFS was associated with higher daily fruit/vegetable intake. In addition, controlling for cancer history, quality of psychological health was associated with physical activity level (*β* = 0.41, *p* = .043, 95% CI 0.01–0.80, adjusted *R*^2^ = 0.17), such that AYAs who reported engaging in more physical activity reported better quality of psychological health.

### Mixed methods results

Our third aim was to integrate and evaluate congruence between qualitative and quantitative findings. The joint display (see Table 1) uses the CSM as an organizing framework to link qualitative themes to congruent items and measures from relevant survey domains, including illness representations, LFS coping strategies, and HRQOL. Illustrative quotes were included to clarify and validate findings that converged in qualitative and quantitative data.

**Table 1:** Joint display of diet and physical activity behaviors and psychosocial associations

CSM concepts	Themes and properties	Measures	Mean (SD)	Illustrative quotes	
Illness representations	Striving for a sense of control over health • LFS/cancer worry may drive control-seeking and heightened monitoring of diet and physical activity behaviors surrounding the time of diagnosis • With time, may appraise level of control over health vs. cancer; influenced by personal and family LFS/cancer consequences • Striving to balance diet and physical activity with life enjoyment vs. overcontrolling behaviors	Worry: LFS and/or cancer risk worry Control: I feel like I have control over my LFS. Consequences: LFS affects my life.	3.72 (0.94)^a^ 2.81 (1.10)^a^ 3.81 (1.02)^a^	• “After the initial (LFS) diagnosis, I think I was really scared… I don’t even think I would eat cheese…” (worry) • “I’m very, very much in control of my health, but very out of control of my cancer issue.” (control) • “I know that there are things you can do, but then I also know that it’s [cancer] totally random…” (consequences) • “I got re-diagnosed doing quote, unquote, everything right.” (consequences)	
Coping strategies and health outcomes	Engaging in healthy diet and physical activity behaviors to protect HRQOL • Viewed as ways to improve HRQOL by: o Helping the body prepare for/protect from future cancers o Keeping strong during treatments and facilitating rehabilitation o Reducing stress, anxiety, depression o Distracting from difficult emotions • Behavior change difficult for some due to internal or external factors (e.g., low motivation, social norms) • Deriving the benefits of health behaviors challenged by treatment-related changes	Daily fruit/veg intake (score) Level of physical activity (min/week) Active coping Quality of physical health Quality of psychological health	7.51 (2.57)^b^ 129.64 (94.08)^c^ 2.47 (0.92)^d^ 65.78 (18.25)^e^ 67.54 (21.74)^e^	• “Before I was diagnosed, I… ate whatever I want. Now, I try to… have a balanced diet.” • “It’s really true that exercising is my mental health pill.” • “I feel like most of the time yoga’s really good and relaxing for me. And then sometimes, I end up leaving really mad and upset about all the ways my body feels like it’s failed me.”	

^a^ 1 = disagree strongly, 2 = disagree, 3 = neither disagree nor agree, 4 = agree, and 5 = agree strongly; ^b^ 1 = no daily fruits/vegetables, 2 = 1/2 cup or less, 3 = 1/2 − 1 cup, 4 = 1–2 cups, 5 = 2–3 cups, 6 = 3–4 cups, and 7 = 4 or more cups; ^c^ Reported number of weekly minutes of at least moderate-intensity physical activity; ^d^ I haven’t been doing this at all, 2 = I’ve been doing this a little, 3 = I’ve been doing this a medium amount, 4 = I’ve been doing this a lot; ^e^ Range = 0-100 on WHOQOL-BREF (World Health Organization, 1998)

Overall, we found convergence in the qualitative and quantitative data regarding illness representations. For example, in interviews, AYAs often described substantial cancer worry, limited personal control over LFS, and a multitude of negative physical and psychosocial consequences of LFS. These qualitative findings were corroborated in survey data in that, on average, AYAs indicated they “agree” that they are worried about LFS/cancer risk, were neutral (“neither disagree nor agree”) that they have personal control of LFS, and “agree” that LFS affects their life. Regarding LFS coping strategies and HRQOL, qualitative data showed that healthy diet and physical activity may serve as active coping strategies, with mostly beneficial effects on HRQOL. The benefit of engaging in these behaviors, however, was sometimes challenged by control beliefs, shifting values, and functional health losses. These qualitative findings may help explain quantitative data, which showed overall modest average levels of reported daily fruit/vegetable intake and physical activity, with wide variation across the sample.

## Discussion

### Principal findings

Our findings suggest AYAs may be motivated to adopt health-protective behaviors in response to an LFS or LFS-related cancer diagnosis, such as eating more healthfully and increasing physical activity. Despite high lifetime genetic cancer risk, most AYAs were not fatalistic about cancer or unmotivated to adopt healthier behaviors as some have posited may occur among individuals with high genetic risk of cancer (Marteau & Weinman, 2006). Our results demonstrate that the situation is more complex. In our sample, AYAs’ healthy diet and physical activity behaviors appeared to function as strategies to gain a sense of control over health (even if it did not prevent cancer) and to strengthen physical and psychological health along the trajectory of genetic illness, including prior to a cancer diagnosis. Eating healthfully and being physically active were ways AYAs could actively cope day-to-day with the burden of LFS/cancer worry and the negative physical and psychosocial consequences of living with an inherited cancer syndrome and, for many, multiple primary cancers. Healthy diet and physical activity behaviors may be important complements to routine multi-organ cancer screenings and risk-reducing procedures (e.g., mastectomy) to protect multiple domains of health, including psychological health. In addition, they may be key targets for future behavioral intervention research.

AYAs often reported that they adjusted their health behaviors (and reframed thoughts about those behaviors) to align them with what they valued at any given time. Values described frequently in this AYA population included prioritizing life enjoyment, reducing stress rather than amplifying it with restrictive health behaviors, feeling confident they had done everything they could to protect their health, and maintaining their health identity. Past research of adults with Lynch syndrome corroborates our finding that life enjoyment and feeling in control of rather than controlled by one’s genetic illness is valued in the context of high cancer risk, and that these values influence health behaviors (Visser et al., 2017).

Despite AYAs’ reported motivation to eat healthfully and be physically active, only about half indicated levels of daily fruit intake that met American Cancer Society recommendations (Rock et al., 2020, 2022). The proportion of AYAs who met daily vegetable intake and weekly aerobic physical activity recommendations was even smaller, under one third. Compared with a nationally representative sample of AYA cancer survivors (Skiba et al., 2021), AYAs with LFS met recommendations at a lower rate for fruit (46% vs. 67%) and vegetable intake (32% vs. 46%) and a similar rate for aerobic physical activity (29% vs. 31%). These results should be interpreted with caution, however, given our very small sample size and potential lack of representativeness (e.g., predominantly college educated, White, non-Hispanic). Studies of larger and more representative samples of AYAs with hereditary cancer syndromes are needed to validate these findings.

### Using diet and physical activity behaviors to cope with a hereditary cancer syndrome

In interviews, several AYAs mentioned that healthy diet and physical activity behaviors function as strategies to cope with syndrome-related health challenges, such as distress, anxiety, and depression. This qualitative finding was supported, in part, by survey data. More frequent use of active LFS coping strategies was associated with healthier behaviors; however, this was only the case for higher reported levels of daily fruit/vegetable intake. Although we did not find a significant relationship between use of active LFS coping strategies and level of physical activity, this may have been due to insufficient power. Notably, in addition to active coping, acceptance, self-distraction, and planning were reported with a relatively high frequency in survey data. Future studies of AYAs could explore the meaning and usefulness of these coping strategies in the context of LFS.

We also found that more frequent use of humor to cope with LFS was associated with higher levels of daily fruit/vegetable intake. Humor may be an adaptive way to cope with stressful situations and reduce anxiety when a situation, such as cancer risk, feels out of a person’s control (Shapiro et al., 2010). Past studies of cancer survivors have also found a positive relationship between active efforts to reduce stress (e.g., using humor) and preventive health behaviors (Parelkar et al., 2013). Additionally, nutrition behavior has been empirically linked to optimism (Soliah, 2011), which has robust associations with better cancer-related physical and psychological health outcomes (Fasano et al., 2020). Thus, in the current research, humor could be acting as a proxy variable for another, third variable.

Our study extends the literature on the favorable impact of healthy diet and physical activity on HRQOL in the general population and among cancer survivors (Rock et al., 2020, 2022) as we found that these behaviors may also support AYAs’ perceived quality of health while living with high, lifelong, multi-organ cancer risk associated with LFS. In interviews, several AYAs shared that healthy diet and physical activity helped them prepare for or protect from future cancers, kept them physically strong during and after treatments, reduced their stress, anxiety, and depression, and helped them cope with difficult thoughts and emotions. Thematic findings did not differ substantively among those with versus without a prior cancer history; rather, perceived health benefits and/or challenges of diet and physical activity behaviors appeared to vary by psychological and/or physical adjustment to LFS, LFS-related procedures (i.e., mastectomy), and cancer(s). Pre-cancer and survivorship care that involves diet and physical activity education and psychological counseling may be valuable for young people with hereditary cancer syndromes given that high risk of medical and psychosocial sequelae can adversely affect their health for long periods of the lifespan.

Overall, average scores for AYAs’ perceived quality of physical health and psychological health were similar to those of older aged survivors of breast cancer off active treatment (Valenti et al., 2008). We found that higher reported levels of physical activity were associated with better perceived quality of psychological health. Future studies with larger samples of AYAs with hereditary cancer syndromes could investigate whether diet or physical activity are associated with perceived quality of physical health to clarify whether the nonsignificant association we found was due to insufficient power or other factors.

Though AYAs mentioned several perceived benefits of healthy diet and physical activity behaviors in interviews, they also shared that these health behaviors sometimes introduce new and evolving stressors. Challenges they identified included fatigue and functional limitations, self-judgment, and feeling psychologically controlled by health behaviors. The impact of diet and physical activity behaviors on HRQOL may, therefore, need frequent self-appraisal and adaptability along the illness trajectory, which may add to the burden of LFS. Support from clinicians and family may be especially important when such challenges arise.

We did not find significant associations between diet and physical activity behaviors and illness representations. Literature conflicts with respect to associations among these variables (Durazo & Cameron, 2019; Ferrer et al., 2018; Séguin Leclair et al., 2021). Given our small sample size and inconclusive literature, we recommend further investigation in larger samples. We did, however, find a significant negative association between LFS/cancer risk worry (emotional illness representations) and perceived control of LFS (see Electronic Supplementary Material 4 Table S3). These results suggest that LFS/cancer risk worry may be an important factor to consider with respect to AYAs’ health beliefs and behaviors. Past research has shown that when stress is high and chronic and emotion regulation is poor, health-promoting behaviors may be compromised (Ferrer et al., 2018). AYAs with syndrome-related worry may benefit from supportive care interventions that address this worry (e.g., through mental health counseling) in tandem with providing health behavior education and counseling.

### Strengths and limitations

This study examined a neglected area of research in young people with inherited multi-organ cancer risk and conclusions that can be drawn from our findings were strengthened by our mixed-methods approach. Our quantitative analysis was limited by self-reported, cross-sectional survey data from which we cannot infer causality; however, our overall findings were enriched by our ability to triangulate survey data with qualitative interview data. We recommend future studies include more comprehensive measures of diet and physical activity as few items and missing physical activity data limited quantitative analysis. In addition, we recommend that future studies measure other LFS coping strategies that AYAs reported using in interview data and that may have important effects on behavioral and psychological health, such as positive reframing.

Although our sample size was small, particularly for quantitative analysis, we anticipated this barrier given that LFS is rare, and we included an age-limited subset of the LFS population. Our sample was also limited by being predominantly female, racially homogeneous, and comprised of mostly young adults (i.e., few adolescents). In addition, due to protocol-mandated annual cancer screening visits, this study cohort may have differed from non-participants.

### Clinical and research implications

Current diet and physical activity recommendations for cancer risk reduction and healthy survivorship does not account for the complex and dynamic health status of individuals with inherited multi-organ cancer risk or the unique needs of young people. Given the health behavior challenges some AYAs described, clinical care is needed that incorporates early and ongoing personalized, values-based diet and physical activity guidance and counseling. Specifically, our study findings suggest that AYAs may benefit from anticipatory guidance provided by their health care team regarding how to support a balanced relationship with diet and physical activity to increase feeling in control of their health rather than feeling controlled by their health behaviors. In counseling, clinicians might focus on nourishment and physical movement as self-care to improve quality of physical and psychological health. Clinicians might emphasize the value of self-compassion versus self-judgment and validate AYAs efforts toward improving overall health by engaging in healthy behaviors.

Clinicians might also keep in mind when discussing diet and physical activity that AYAs may be at various stages of readiness for behavior change, ranging from uninterested to engaging in practices that are potentially harmful (e.g., disordered eating, compulsive exercise). This is particularly critical to consider in the AYA population given compounding social and syndrome-related stressors as well as their vulnerability and easy access to abundant cancer nutrition misinformation online as frequent internet users (Mooney et al., 2016; Warner et al., 2022). AYAs (and their family members) may especially benefit from anticipatory guidance regarding how to find credible information about diet for cancer risk reduction and healthy survivorship. Referral to a registered dietitian nutritionist and/or mental health counselor who specializes in oncology may be essential for some AYAs.

In addition to including more comprehensive measures of diet and physical activity, future studies of AYAs with hereditary cancer syndromes could include measures of behavioral self-efficacy, which we did not assess in our survey. The literature would also be strengthened by longitudinal studies that evaluate health behavior change over time. Supportive care intervention research that includes diet and physical activity guidance and counseling tailored to AYAs with hereditary cancer syndromes is needed. Working in partnership with AYAs from the study design phase may ensure such interventions are focused on outcomes meaningful to AYAs.

## Conclusions

Healthy diet and physical activity behaviors were viewed by AYAs as active ways to cope with a hereditary cancer syndrome, LFS. Most believed that their overall health and cancer risk are somewhat controllable through these health behaviors. Results suggest engaging in regular physical activity may especially have mental health benefits for AYAs with LFS, many of whom report experiencing mental health challenges due to their genetic condition. Clinicians might support AYAs in adopting diet and physical activity behaviors that protect multiple aspects of health (e.g., mental, nutritional). Future research could focus on developing AYA- and syndrome-specific supportive care interventions that include both behavioral and mental health counseling.

## Electronic Supplementary Material

Below is the link to the electronic supplementary material.

Supplementary Material 1

Supplementary Material 2

Supplementary Material 3

Supplementary Material 4

Supplementary Material 5

Supplementary Material 6

## Data Availability

De-identified data from this study are not available in a public archive. De-identified data from this study will be made available (as allowable according to the National Institutes of Health IRB standards) by emailing the corresponding author. Materials used to conduct this study are not publicly available.

## Code Availability

Analytic code used to conduct the analyses presented in this study are not available in a public archive. They may be available by emailing the corresponding author.
