# Supplementary material for: Diet and physical activity behaviors: how are they related to illness perceptions, coping, and health-related quality of life in young people with hereditary cancer syndromes?
Source: J Behav Med. 2024 Apr 20;47(4):707–20. doi: 10.1007/s10865-024-00489-z (PMC11291531; doi:10.1007/s10865-024-00489-z)
Supplement: Supplementary file 1 — Supplementary Material 1 [file 10865_2024_489_MOESM1_ESM.pdf]

Electronic Supplementary Material 1

Interview Questions

Wave 1

Select questions relevant to the current study:

1. What is your perspective on cancer prevention? Have you engaged in any specific behaviors to help lower your risk of cancer?
2. Are there any things, big or small, that you do every day that help you feel good—emotionally, physically, and/or both? Do(es) LFS, (if applicable: your cancer diagnosis, or your treatment) impact these activities at all? If so, how? Does LFS impact anything you can't do or wish to do?
3. How much control do you feel you have over LFS and cancer? In what ways? Has this changed over time? Are there places where you feel more or less control?

Wave 2

Select questions relevant to the current study:

Interviewer: I want to check in to see how you are feeling and how LFS may or may not be impacting your daily life and activities, particularly in light of the COVID-19 pandemic.

1. Tell me how the COVID-19 pandemic has changed your daily life (*e.g.,* doing enjoyed activities more or less [interviewer note: if relevant, reflect on activities reported during the wave 1 interview to assess any changes])?
2. Tell me more about activities that are most important to you. How do you benefit from them? This goes for activities that you've adopted since the COVID-19 pandemic and activities you've been doing for a long time. What purpose do they serve? What do you enjoy about them? (*e.g.,* distraction, self-care, connection to others, pursuing a goal)
3. If relevant, what do you miss about the activities that you have had to stop during the COVID-19 pandemic?
