# Supplementary material for: Diet and physical activity behaviors: how are they related to illness perceptions, coping, and health-related quality of life in young people with hereditary cancer syndromes?
Source: J Behav Med. 2024 Apr 20;47(4):707–20. doi: 10.1007/s10865-024-00489-z (PMC11291531; doi:10.1007/s10865-024-00489-z)
Supplement: Supplementary file 2 — Supplementary Material 2 [file 10865_2024_489_MOESM2_ESM.pdf]

Electronic Supplementary Material 2

Table S1.

Participant Demographics

	Wave 1	*Wave 2*		*Total*
Demographic	Interview (*n*=38)	Interview (*n*=30)	Survey (*n*=37)	All samples (*n*=57) [a]
Age (years)				
Mean (SD)	29 (6.5)	31 (6.2)	31 (6.0)	30 (6.5)
Range	17-40	18-41	19-39	17-40
Age at genetic testing (years) [b]				
Mean (SD)	22 (7.8)	23 (6.9)	24 (6.3)	23 (7.5)
Range	1-35	2-35	12-35	1-35
Gender (*n*, %)				
Male	12 (32)	9 (30)	7 (19)	15 (26)
Female	26 (68)	21 (70)	29 (78)	41 (72)
Genderqueer	0 (0)	0 (0)	1 (3)	1 (2)
Race [c] (*n*, %)				
Asian	0 (0)	1 (3)	2 (5)	2 (4)
White	32 (84)	26 (87)	32 (86)	49 (86)
Multiracial	5 (12)	2 (7)	3 (8)	5 (9)
American Indian/Alaska Native and White	1 (3)	0 (0)	1 (3)	1 (2)
Asian and White	1 (3)	1 (3)	1 (3)	1 (2)
Black/African American and White	2 (5)	1 (3)	0 (0)	2 (4)
Native Hawaiian/Other Pacific Islander and White	1 (3)	0 (0)	1 (3)	1 (2)
Missing	1 (3)	1 (3)	0 (0)	1 (2)
Ethnicity (*n*, %)				
Hispanic/Latino	2 (5)	2 (7)	2 (5)	3 (5)
Non-Hispanic/Latino	35 (92)	27 (90)	35 (95)	53 (93)
Missing	1 (3)	1 (3)	0 (0)	1 (2)
Educational attainment [d] (*n*, %)				
Attending high school	2 (5)	1 (3)	0 (0)	2 (4)
Attending college	2 (5)	1 (3)	0 (0)	2 (4)
Non-college graduate	8 (21)	9 (30)	10 (27)	13 (23)
College graduate	18 (47)	12 (40)	27 (73)	32 (56)
Missing	8 (21)	7 (23)	0 (0)	8 (14)
Cancer status				
No cancer (*n*, %)	15 (39)	10 (33)	18 (49)	25 (44)
≥ 1 primary cancer (*n*, %)	23 (61)	20 (67)	19 (51)	32 (56)
Age at first primary cancer diagnosis (SD) (years)	20 (10.6)	22 (9.4)	23 (9.4)	22 (9.8)

Range (years)	0.5-35	0.5-35	0.5-35	0.5-35

[a] Samples are not independent; participants can be counted multiple times across data collection points.

[b] Missing data: n=3

[c] Participants could report multiple races; totals may exceed 100%.

[d] Non-college graduates include those whose highest educational attainment is a high school diploma/GED or post-high school training (technical or vocational school). College graduates include those whose highest educational attainment is an associate, bachelor's, or graduate degree.
