# Supplementary material for: Diet and physical activity behaviors: how are they related to illness perceptions, coping, and health-related quality of life in young people with hereditary cancer syndromes?
Source: J Behav Med. 2024 Apr 20;47(4):707–20. doi: 10.1007/s10865-024-00489-z (PMC11291531; doi:10.1007/s10865-024-00489-z)
Supplement: Supplementary file 3 — Supplementary Material 3 [file 10865_2024_489_MOESM3_ESM.pdf]

**Electronic Supplementary Material 3**

**Table S2.**

*Descriptive Statistics for Study Variables*

| Variable | *M* | *SD* | *Median* | *Range* |
|---|---|---|---|---|
| Daily fruit and vegetable intake score [a] | 7.51 | 2.57 | 7 | 4-14 |
| Daily fruit intake score | 3.54 | 1.35 | 3 | 2-7 |
| Daily vegetable intake score | 3.97 | 1.54 | 4 | 1-7 |
| Physical activity minutes per week (n = 28) | 129.64 | 94.08 | 105 | 20-315 |
| Concern about LFS and/or cancer risk (n = 36) [b] | 3.72 | 0.94 | 4 | 1-5 |
| Cognitive illness representations [b] | | | | |
| LFS affects my life. | 3.81 | 1.02 | 4 | 1-5 |
| I feel like I have control over my LFS. | 2.81 | 1.10 | 3 | 1-5 |
| Emotional illness representations (α=.85) (n = 36) [b] | 3.72 | 0.94 | 4 | 1-5 |
| LFS coping strategies [c] | | | | |
| Self-distraction | 2.55 | 1.03 | 3 | 1-4 |
| Emotional support | 2.36 | 0.92 | 2.50 | 1-4 |
| Instrumental support | 2.03 | 0.94 | 2 | 1-4 |
| Venting | 2.03 | 0.90 | 2 | 1-4 |
| Planning | 2.45 | 1.04 | 2.50 | 1-4 |
| Humor | 1.82 | 0.90 | 1.50 | 1-4 |
| Acceptance | 2.91 | 1.05 | 3 | 1-4 |
| Religion | 2.15 | 1.07 | 2 | 1-4 |
| Active coping (n = 36) | 2.47 | 0.92 | 2.50 | 1-4 |
| Health-related quality of life [d] | | | | |
| Physical health (α=.86) | 67.54 | 21.74 | 69 | 6-100 |
| Psychological health (α=.86) | 65.78 | 18.25 | 69 | 6-94 |

[a] 1 = no daily fruits/vegetables, 2 = 1/2 cup or less, 3=1/2 cup to 1 cup, 4 = 1 to 2 cups, 5 = 2 to 3 cups, 6 = 3 to 4 cups, and 7 = 4 or more cups.

[b] 1=disagree strongly, 2=disagree, 3=neither disagree nor agree, 4=agree, and 5=agree strongly

[c] 1 = I haven’t been doing this at all, 2 = I’ve been doing this a little, 3 = I’ve been doing this a medium amount, 4 = I’ve been doing this a lot

[d] Range = 0-100, with higher scores indicating better quality of health for each domain (*i.e.*, physical, psychological)
