# Supplementary material for: Diet and physical activity behaviors: how are they related to illness perceptions, coping, and health-related quality of life in young people with hereditary cancer syndromes?
Source: J Behav Med. 2024 Apr 20;47(4):707–20. doi: 10.1007/s10865-024-00489-z (PMC11291531; doi:10.1007/s10865-024-00489-z)
Supplement: Supplementary file 4 — Supplementary Material 4 [file 10865_2024_489_MOESM4_ESM.pdf]

Electronic Supplementary Material 4

Table S3.

Correlations Between Study Variables

	1	2	3	4	5	6	7	8	9	10	11	12	13	14	15	16
1. Daily Fruit and Vegetable Intake [a]	-															
2. Physical Activity Minutes Per Week (n = 28) [a]	.19	-														
3. LFS affects my life [a]	-.03	-.05	-													
4. I feel like I have control over my LFS [a]	.10	.17	-.16	-												
5. Emotional Illness Representations [a]	-.15	.17	.77**	-.32	-											
6. Coping: Self-Distraction [a]	.26	.06	.20	-.02	.04	-										
7. Coping: Emotional Support [a]	.14	-.01	.30	-.14	.26	.56**	-									
8. Coping: Instrumental Support [a]	.04	-.16	.16	-.14	.11	.50**	.83**	-								
9. Coping: Venting [a]	.19	.06	.37*	-.28	.45**	.46**	.75**	.61**	-							
10. Coping: Planning [a]	.08	-.20	.33*	-.13	.20	.52**	.78**	.75**	.66**	-						
11. Coping: Humor [a]	.37*	-.19	.05	.10	-.10	.38*	.31	.25	.20	.34	-					
12. Coping: Acceptance [a]	.09	-.18	.32	.23	.17	.51**	.49**	.54**	.36*	.71**	.43*	-				
13. Coping: Active Coping (n = 36) [a]	.34*	-.04	.11	.16	-.09	.55	.60**	.55**	.49**	.63**	.51**	.55**	-			
14. Physical Health [a]	.18	.40*	-.42*	.59**	-.54**	-.17	-.24	-.29	-.43**	-.20	-.16	-0.12	-.01	-		
15. Psychological Health [a]	.16	.40*	-.08	.46**	-.34	.10	.06	-.03	-.21	.10	-.00	.14	.16	.76**	-	
16. Cancer History [b]	.01	.31	.25	-.02	.30	-.14	.12	-.03	.12	.11	.11	-.04	0	.10	.08	-

*p < .05; **p < .01

[a] Bivariate correlations were calculated.

[b] Point biserial correlations were calculated.
