# Supplementary material for: Diet and physical activity behaviors: how are they related to illness perceptions, coping, and health-related quality of life in young people with hereditary cancer syndromes?
Source: J Behav Med. 2024 Apr 20;47(4):707–20. doi: 10.1007/s10865-024-00489-z (PMC11291531; doi:10.1007/s10865-024-00489-z)
Supplement: Supplementary file 5 — Supplementary Material 5 [file 10865_2024_489_MOESM5_ESM.pdf]

Electronic Supplementary Material 5

Table S4.

Regression Coefficients for Predicting Fruit and Vegetable Intake from "LFS affects my life"

Variable	*Estimate*	*SE*	*t value*	*p value*	*95% CI*
Intercept	0	.17	.00	1.00	-.34, .34
LFSpercept_1	-0.03	.18	-.16	.873	-.39, .33
Cancer History	.01	.18	.07	.945	-.35, .37

Table S5.

Regression Coefficients for Predicting Fruit and Vegetable Intake from "I feel like I have control over my LFS"

Variable	*Estimate*	*SE*	*t value*	*p value*	*95% CI*
Intercept	0	.17	.00	1.00	-.34, .34
LFSpercept2	.10	.17	.61	.545	-.24, .45
Cancer History	.01	.17	.04	.966	-.34, .35

Table S6.

Regression Coefficients for Predicting Fruit and Vegetable Intake from Emotional Illness Representations

Variable	*Estimate*	*SE*	*t value*	*p value*	*95% CI*
Intercept	-.04	.17	-.24	.810	-.38, .30
Illness Representations	-.18	.18	-1.00	.327	-.54, .18
Cancer History	.10	.18	.55	.584	-.26, .46

Table S7.

Regression Coefficients for Predicting Fruit and Vegetable Intake from Coping: Self-Distraction

Variable	*Estimate*	*SE*	*t value*	*p value*	*95% CI*
Intercept	.00	.16	.00	1.00	-.33, .33
Self-Distraction	.27	.17	1.61	.116	-.07, .61
Cancer History	.04	.17	.25	.805	-.30, .38

Table S8.

Regression Coefficients for Predicting Fruit and Vegetable Intake from Coping: Emotional Support

Variable	*Estimate*	*SE*	*t value*	*p value*	*95% CI*
Intercept	0	.17	.00	1.00	-.34, .34
Emotional Support	.14	.17	.81	.426	-.21, .49
Cancer History	-.01	.17	-.07	.945	-.36, .34

Table S9.

Regression Coefficients for Predicting Fruit and Vegetable Intake from Coping: Instrumental Support

Variable	*Estimate*	*SE*	*t value*	*p value*	*95% CI*
Intercept	0	.17	.00	1.00	-.34, .34
Instrumental Support	.04	.17	.24	.816	-.31, .39
Cancer History	.01	.17	.04	.970	-.34, .35

Table S10.

Regression Coefficients for Predicting Fruit and Vegetable Intake from Coping: Venting

Variable	*Estimate*	*SE*	*t value*	*p value*	*95% CI*
Intercept	0	.17	.00	1.00	-.34, .34
Venting	.19	.17	1.11	.273	-.16, .53
Cancer History	-.02	.17	-.11	.917	-.36, .33

Table S11.

Regression Coefficients for Predicting Fruit and Vegetable Intake from Coping: Planning

Variable	*Estimate*	*SE*	*t value*	*p value*	*95% CI*
Intercept	0	.17	.00	1.00	-.34, .34
Planning	.08	.17	.49	.628	-.27, .43
Cancer History	-.00	.17	-.02	.983	-.35, .35

Table S12.

Regression Coefficients for Predicting Fruit and Vegetable Intake from Coping: Humor

Variable	*Estimate*	*SE*	*t value*	*p value*	*95% CI*
Intercept	0	.16	.00	1.00	-.32, .32
Humor	.37	.16	2.34	.025	.05, .70
Cancer History	-.04	.16	-.23	.823	-.36, .29

Table S13.

Regression Coefficients for Predicting Fruit and Vegetable Intake from Coping: Acceptance

Variable	*Estimate*	*SE*	*t value*	*p value*	*95% CI*
Intercept	0	.17	.00	1.00	-.34, .34
Acceptance	.09	.17	.50	.618	-.26, .43
Cancer History	.01	.17	.05	.961	-.34, .36

Table S14.

Regression Coefficients for Predicting Fruit and Vegetable Intake from Coping: Active Coping

Variable	*Estimate*	*SE*	*t value*	*p value*	*95% CI*
Intercept	-.01	.16	-.03	.975	-.34, .33
Active Coping	.34	.17	2.04	.049	.00, .68
Cancer History	-.00	.17	.00	1.00	-.34, .34

Table S15.

Regression Coefficients for Predicting Fruit and Vegetable Intake from Physical Health

Variable	*Estimate*	*SE*	*t value*	*p value*	*95% CI*
Intercept	.00	.17	.00	1.00	-.34, .34
Physical Health	.18	.17	1.06	.298	-.17, .52
Cancer History	-.01	.17	-.07	.943	-.36, .33

Table S16.

Regression Coefficients for Predicting Fruit and Vegetable Intake from Psychological Health

Variable	*Estimate*	*SE*	*t value*	*p value*	*95% CI*
Intercept	0.00	.17	.00	1.00	-.34, .34
Psychological Health	.16	.17	.94	.354	-.18, .50
Cancer History	-.01	.17	-.04	.965	-.35, .34
