# Supplementary material for: Diet and physical activity behaviors: how are they related to illness perceptions, coping, and health-related quality of life in young people with hereditary cancer syndromes?
Source: J Behav Med. 2024 Apr 20;47(4):707–20. doi: 10.1007/s10865-024-00489-z (PMC11291531; doi:10.1007/s10865-024-00489-z)
Supplement: Supplementary file 6 — Supplementary Material 6 [file 10865_2024_489_MOESM6_ESM.pdf]

Electronic Supplementary Material 6

Table S17.

Regression Coefficients for Predicting Physical Activity from "LFS affects my life"

Variable	*Estimate*	*SE*	*t value*	*p value*	*95% CI*
Intercept	.00	.19	.00	.999	-.39, .39
LFSpercept1	-.10	.21	-.45	.656	-.53, .34
Cancer History	.32	.19	1.68	.105	-.07, .71

Table S18.

Regression Coefficients for Predicting Physical Activity from "I feel like I have control over my LFS"

Variable	*Estimate*	*SE*	*t value*	*p value*	*95% CI*
Intercept	-.04	.18	-.20	.846	-.42, .34
LFSpercept2	.21	.20	1.02	.319	-.21, .62
Cancer History	.32	.19	1.73	.096	-.06, .71

Table S19.

Regression Coefficients for Predicting Physical Activity from Emotional Illness Representations

Variable	*Estimate*	*SE*	*t value*	*p value*	*95% CI*
Intercept	-.04	.19	-.19	.852	-.44, .36
Illness Representations	.11	.21	.62	.608	-.33, .55
Cancer History	.30	.20	1.50	.146	-.11, .72

Table S20.

Regression Coefficients for Predicting Physical Activity from Coping: Self-Distraction

Variable	*Estimate*	*SE*	*t value*	*p value*	*95% CI*
Intercept	-.02	.19	-.13	.897	-.41, .36
Self-Distraction	.13	.19	.71	.486	-.26, .52
Cancer History	.34	.19	1.77	.089	-.06, .74

Table S21.

Regression Coefficients for Predicting Physical Activity from Coping: Emotional Support

Variable	*Estimate*	*SE*	*t value*	*p value*	*95% CI*
Intercept	-.01	.19	-.07	.942	-.41, .38

Emotional Support	.00	.21	.01	.993	-.42, .43
Cancer History	.31	.19	1.63	.115	-.08, .70

Table S22.

Regression Coefficients for Predicting Physical Activity from Coping: Instrumental Support

Variable	*Estimate*	*SE*	*t value*	*p value*	*95% CI*
Intercept	0.00	.19	.01	.992	-.39, .39
Instrumental Support	-.10	.19	-.53	.602	-.50, .30
Cancer History	.29	.19	1.51	.143	-.11, .69

Table S23.

Regression Coefficients for Predicting Physical Activity from Coping: Venting

Variable	*Estimate*	*SE*	*t value*	*p value*	*95% CI*
Intercept	-.02	.19	-.10	.921	-.41, .37
Venting	.04	.20	.21	.832	-.36, .45
Cancer History	.31	.19	1.61	.119	-.08, .70

Table S24.

Regression Coefficients for Predicting Physical Activity from Coping: Planning

Variable	*Estimate*	*SE*	*t value*	*p value*	*95% CI*
Intercept	.01	.19	.07	.949	-.37, .39
Planning	-.19	.19	-.97	.342	-.57, .20
Cancer History	.30	.19	1.60	.122	-.09, .68

Table S25.

Regression Coefficients for Predicting Physical Activity from Coping: Humor

Variable	*Estimate*	*SE*	*t value*	*p value*	*95% CI*
Intercept	-.03	.19	-.14	.894	-.41, .36
Humor	-.15	.20	-.77	.448	-.56, .26
Cancer History	.29	.19	1.62	.141	-.10, .68

Table S26.

Regression Coefficients for Predicting Physical Activity from Coping: Acceptance

Variable	*Estimate*	*SE*	*t value*	*p value*	*95% CI*
Intercept	-.01	.19	-.05	.961	-.39, .38
Acceptance	-.08	.22	-.38	.706	-.53, .36
Cancer History	.28	.20	1.40	.174	-.13, .70

Table S27.

Regression Coefficients for Predicting Physical Activity from Coping: Active Coping

Variable	*Estimate*	*SE*	*t value*	*p value*	*95% CI*
Intercept	-.06	.19	-.30	.770	-.44, .33
Active Coping	-.01	.20	-.04	.967	-.41, .39
Cancer History	.27	.19	1.39	.178	-.13, .66

Table S28.

Regression Coefficients for Predicting Physical Activity from Physical Health

Variable	*Estimate*	*SE*	*t value*	*p value*	*95% CI*
Intercept	-.02	.18	-.10	.924	-.38, .34
Physical Health	.34	.19	1.84	.078	-.04, .73
Cancer History	.22	.18	1.21	.238	-.16, .60

Table S29.

Regression Coefficients for Predicting Physical Activity from Psychological Health

Variable	*Estimate*	*SE*	*t value*	*p value*	*95% CI*
Intercept	-.04	.17	-.21	.840	-.39, .32
Psychological Health	.41	.19	2.13	.043	.01, .80
Cancer History	.27	.18	1.55	.135	-.09, .63
